# Hereditary transthyretin amyloid cardiomyopathy caused by the rare TTR p.Ser43Asn variant in an Asian family: a case report

**DOI:** 10.3389/fcvm.2026.1761600

**Published:** 2026-06-08

**Authors:** Bo Song, Youfu He, Xinghui Liu, Hui Liu, Fawang Du, Jiren Wang, Hongwen Tan, Changhai Zhang, Ping Zhang

**Affiliations:** 1 Department of Cardiology, Guizhou Provincial People’s Hospital, Guiyang, Guizhou Province, China; 2 Department of Cardiology, Guizhou Provincial Key Laboratory of Pathogenesis and Prevention of Common Chronic Diseases, Guiyang, Guizhou Province, China

**Keywords:** Asian population, cardiac amyloidosis, case report, hereditary transthyretin amyloidosis, hypertrophic cardiomyopathy mimic, transthyretin amyloid cardiomyopathy, *TTR* c.128G > A (p.Ser43Asn)

## Abstract

**Background:**

Transthyretin amyloid cardiomyopathy (ATTR–CM) is an infiltrative cardiomyopathy that may occur in hereditary or wild-type forms. Although more than 150 transthyretin (*TTR*) variants have been reported, the c.128G > A (p.Ser43Asn) variant is rare, and data from Asian populations remain limited.

**Case presentation:**

This report describes a mainland Chinese family carrying the rare *TTR* c.128G > A (p.Ser43Asn) variant. The proband, a 51-year-old Chinese man, presented with exertional chest tightness and dyspnea. Electrocardiography showed left ventricular hypertrophy, while echocardiography demonstrated concentric left ventricular wall thickening, reduced systolic function, and relative apical sparing on strain imaging. Technetium-99 m pyrophosphate [(99 m)Tc-PYP] scintigraphy showed grade 3 myocardial uptake, and monoclonal protein assessment, including serum and urine immunofixation electrophoresis and serum free light-chain testing, did not support light-chain amyloidosis. Genetic testing identified a heterozygous c.128G > A (p.Ser43Asn) missense variant in the *TTR* gene. Tafamidis was initiated after diagnosis. On follow-up, the proband remained in New York Heart Association functional class II, with persistent structural cardiac abnormalities on echocardiography. Family investigation identified additional affected relatives and carriers of the same variant, supporting a hereditary pattern of disease in this pedigree.

**Conclusion:**

This family report expands the clinical spectrum associated with the rare *TTR* c.128G > A (p.Ser43Asn) variant and adds to the limited data available from Asian populations.

## Introduction

Transthyretin (TTR) cardiac amyloidosis is a restrictive, infiltrative cardiomyopathy caused by the dissociation of unstable TTR tetramers into monomers, which subsequently misfold, aggregate into amyloid fibrils, and deposit in the myocardial interstitium (1). Based on the presence or absence of *TTR* gene variants, transthyretin amyloid cardiomyopathy (ATTR–CM) is classified as either wild-type ATTR–CM (ATTRwt) or variant ATTR–CM (ATTRv) (2–5).

In recent years, major advances in diagnostic techniques and disease-modifying therapies have substantially improved the clinical recognition and management of ATTR–CM. On the one hand, the increasing use of non-invasive imaging and molecular genetic testing has led to the reclassification of many patients who were previously considered to have hypertrophic cardiomyopathy, unexplained left ventricular hypertrophy, or heart failure of uncertain cause as having ATTR–CM (6). On the other hand, the approval of novel therapeutic agents and the continued development of targeted treatments have transformed the clinical landscape of ATTR–CM. A disease once associated with a uniformly poor prognosis is now one in which progression may be significantly delayed with timely diagnosis and intervention (7, 8).

Despite these advances, hereditary ATTR-CM remains highly heterogeneous in both its molecular background and clinical presentation. More than 150 *TTR* variants have been reported to date; 100 are considered clinically significant, exhibiting substantial variation across geographic regions, ethnic groups, and families (9, 10). This distinction helps explain the difference between the total number of reported *TTR* variants and the smaller subset regarded as clinically relevant mutations. In addition, the same variant may elicit markedly different phenotypic manifestations in different individuals (11). Consequently, reports of rare or regionally uncommon *TTR* variants remain highly valuable because they help refine genotype–phenotype correlations and broaden our understanding of disease heterogeneity.

Here, we describe a mainland Chinese family carrying the rare *TTR* c.128G > A (p.Ser43Asn) variant. By synthesizing the proband's clinical presentation, imaging findings, genetic results, and follow-up data with a comprehensive family history, this report expands the phenotypic spectrum associated with this rare variant and adds to the limited data available from Asian populations.

## Case

In 2023, a 51-year-old Chinese man (Patient A) presented with exertional chest tightness and dyspnea, a history of ischemic stroke, and numbness of the left upper limb that had begun 2 months earlier. He also reported decreased superficial sensation, including reduced pain and light-touch sensation, extending from the upper arm to the fingers. No formal neurologic evaluation, nerve conduction study, targeted assessment for carpal tunnel syndrome, or formal evaluation for familial amyloid polyneuropathy was performed. Coronary computed tomography angiography performed at another hospital showed 33% stenosis in the midleft anterior descending artery, with no abnormalities in the remaining coronary arteries. Treatment with aspirin, atorvastatin, and sacubitril/valsartan did not improve his chest tightness or dyspnea.

On physical examination, his blood pressure was 129/67 mmHg. No orthostatic hypotension or cardiac murmur was noted. At baseline, he was in New York Heart Association (NYHA) functional class II. Routine blood, biochemical, and thyroid function test results were largely unremarkable. Serum creatinine was 117 μmol/L, and the estimated glomerular filtration rate (eGFR) was 72 mL/min/1.73 m^2^. Serum protein electrophoresis and serum as well as urine immunofixation electrophoresis were negative, with no monoclonal protein detected. Serum free light-chain testing showed a kappa light chain level of 3.93 g/L, a lambda light chain level of 1.81 g/L, and a kappa/lambda ratio of 2.17. Taken together, these findings did not support a diagnosis of light-chain amyloidosis. Cardiac biomarkers were elevated, with a high-sensitivity troponin I level of 23.83 pg/mL and a B-type natriuretic peptide (BNP) level of 199 pg/mL.

**Figure 1:**
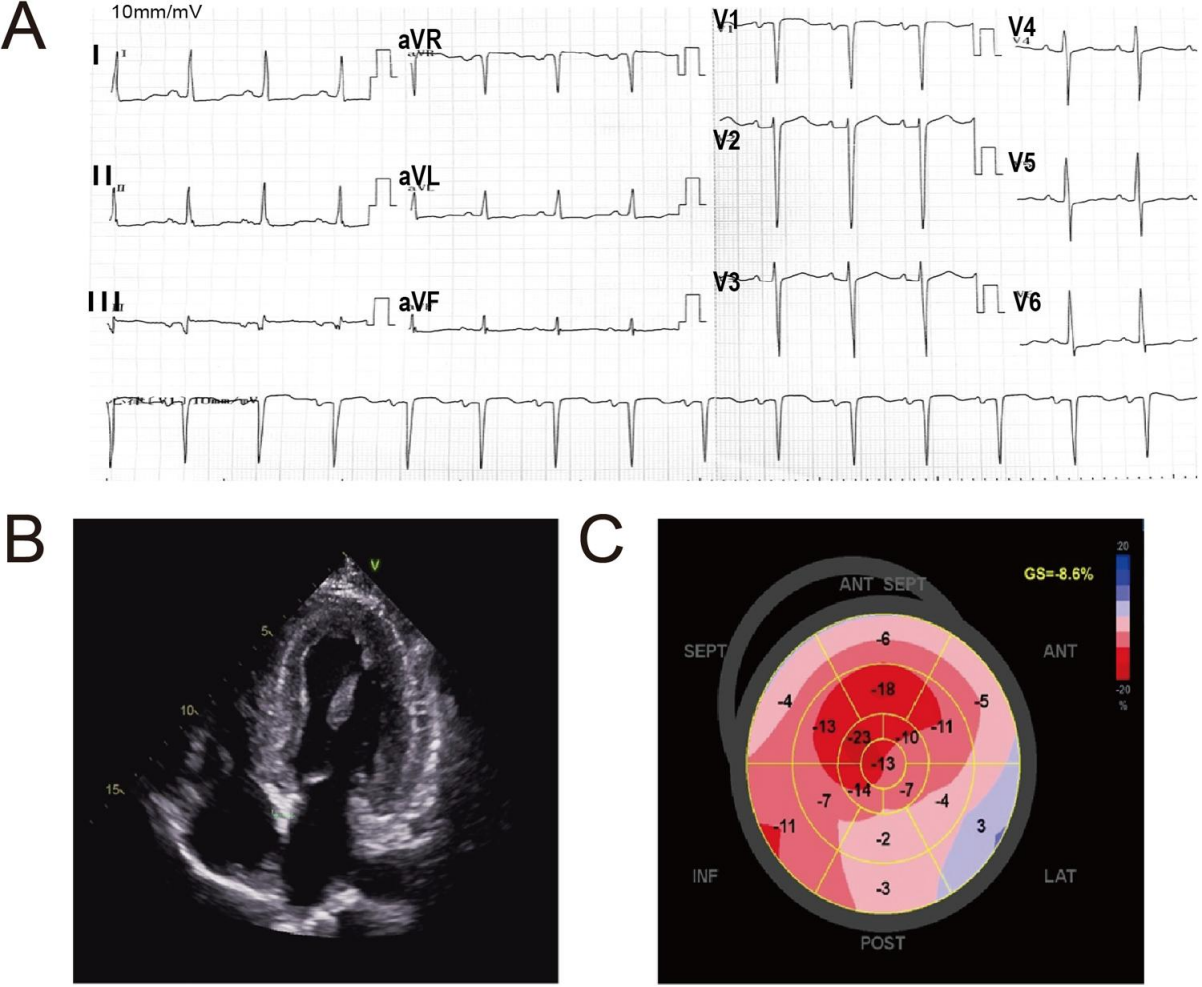
Electrocardiogram, echocardiogram, and left ventricular strain map. **(A)** Electrocardiogram showing left ventricular hypertrophy. **(B)** Echocardiography showed concentric left ventricular wall thickening with a typical granular sparkling appearance of the myocardium, suggestive of amyloid infiltration. **(C)** The left ventricular strain map illustrates the reduced longitudinal strain in the middle and basal segments of the left ventricle, with relatively preserved strain at the apex. The global strain is measured at −8.6%, indicating impaired myocardial function in the affected regions.

**Figure 2:**
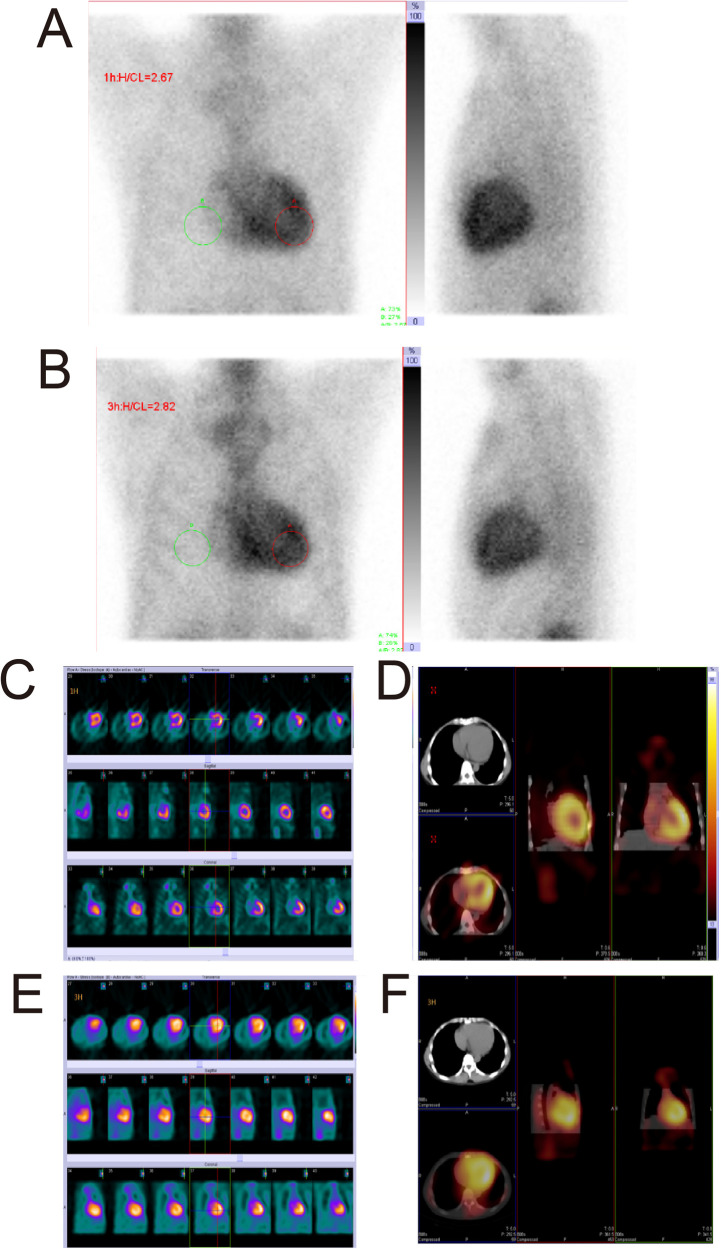
99mTc-PYP myocardial scintigraphy and SPECT/CT fusion at 1 and 3 h demonstrating progressive myocardial uptake. **(A)** Planar ^99mTc-PYP myocardial scintigraphy 1 h after tracer injection (anterior and lateral views) shows mild uptake in the cardiac region, with only weak radiopharmaceutical accumulation. **(B)** At 3 h postinjection, planar anterior and lateral views demonstrate substantial cardiac uptake, with prominent radiopharmaceutical accumulation in the anterior wall. The Perugini visual score is grade 3, indicating a high likelihood of transthyretin amyloid cardiomyopathy (ATTR). **(C)** Transverse, sagittal, and coronal SPECT images at 1 h show mild but definite radiotracer uptake in the cardiac region, suggesting early myocardial amyloid deposition. **(D)** SPECT/CT fusion images at 1 h confirm that the radiotracer uptake is localized to the myocardium, supporting the presence of amyloid deposits. **(E)** Transverse, sagittal, and coronal SPECT images at 3 h reveal markedly increased radiopharmaceutical uptake throughout the cardiac region, with more pronounced accumulation in the myocardial tissue, indicative of more advanced myocardial amyloidosis. **(F)** SPECT/CT fusion images at 3 h highlight intense myocardial uptake of the radiotracer, a pattern consistent with transthyretin amyloid cardiomyopathy.

**Figure 3:**
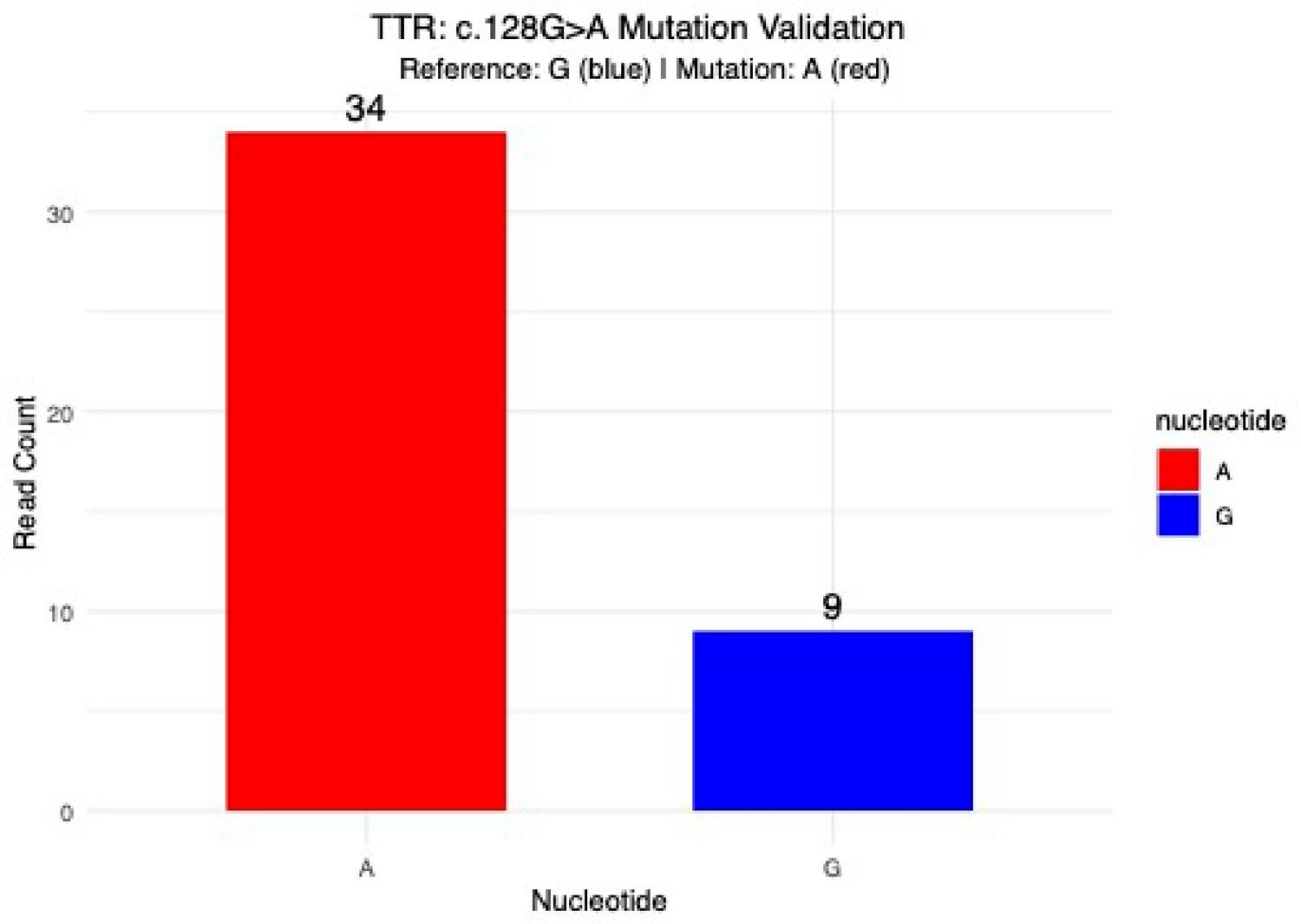
Genetic verification of the c.128G > A (p.Ser43Asn) mutation in the TTR gene. Sequencing results confirm the presence of the c.128G > A (p.Ser43Asn) mutation in the TTR gene, resulting in an amino acid substitution from serine to asparagine at position 43. The data show the ratio of the mutant allele (A) to the wild-type allele (G), with a mutation frequency of approximately 79%, indicating a dominant mutant allele in the patient's genetic profile.

Electrocardiography showed left ventricular hypertrophy (Figure 1A). Echocardiography revealed concentric left ventricular wall thickening with reduced systolic function. The left atrial diameter was 39 mm, the left ventricular ejection fraction was 39%, the fractional shortening was 19%, the interventricular septal thickness was 18 mm, and the E/e′ ratio was >14. A granular sparkling appearance of the myocardium was also noted. Left ventricular longitudinal strain was reduced in the basal and midventricular segments, with relative apical sparing (Figures 1B, C). [99 m]Tc-PYP scintigraphy performed at 1 and 3 h showed marked cardiac uptake, with a Perugini visual score of grade 3, consistent with transthyretin cardiac amyloidosis. In the absence of monoclonal proteins on serum and urine immunofixation electrophoresis, these findings supported a non-biopsy diagnosis of ATTR–CM (Figures 2A–F). Genetic testing identified a heterozygous c.128G > A (p.Ser43Asn) missense variant in the *TTR* gene (Figure 3). This variant has previously been reported in patients with hereditary transthyretin amyloidosis. Following the diagnosis, tafamidis therapy was initiated.

At follow-up in 2024, Patient A remained in NYHA functional class II. Echocardiography continued to show left ventricular wall thickening, with a left atrial diameter of 37 mm, interventricular septal thickness of 15–18 mm, left ventricular posterior wall thickness of 17 mm, left ventricular end-diastolic diameter of 49 mm, ejection fraction of 59%, and fractional shortening of 32%. Tissue Doppler imaging showed a septal e′ of 0.05 m/s, a lateral *e*′ of 0.02 m/s, and an *E/e*′ > 14. Serum creatinine was 122 μmol/L, and the eGFR was 70 mL/min/1.73 m^2^. The high-sensitivity troponin I level was 19.21 pg/mL, and the BNP level was 312 pg/mL. On reassessment in 2025, he remained in NYHA functional class II. Echocardiography showed persistent biatrial enlargement and left ventricular wall thickening, with a left atrial diameter of 43 mm, interventricular septal thickness of 17 mm, left ventricular posterior wall thickness of 17 mm, left ventricular end-diastolic diameter of 51 mm, ejection fraction of 56%, and fractional shortening of 29%. Septal *e*′ was 0.07 m/s, lateral *e*′ was 0.05 m/s, and the *E/e*′ ratio remained >14. Global longitudinal strain was −12.4%, with relative apical sparing. Serum creatinine was 121 μmol/L, and the eGFR was 71 mL/min/1.73 m^2^. The high-sensitivity troponin I level was 17.66 pg/mL, and the BNP level was 188 pg/mL. Serum protein electrophoresis and serum and urine immunofixation electrophoresis remained negative throughout the follow-up period, with no evidence of monoclonal gammopathy.

**Figure 4:**
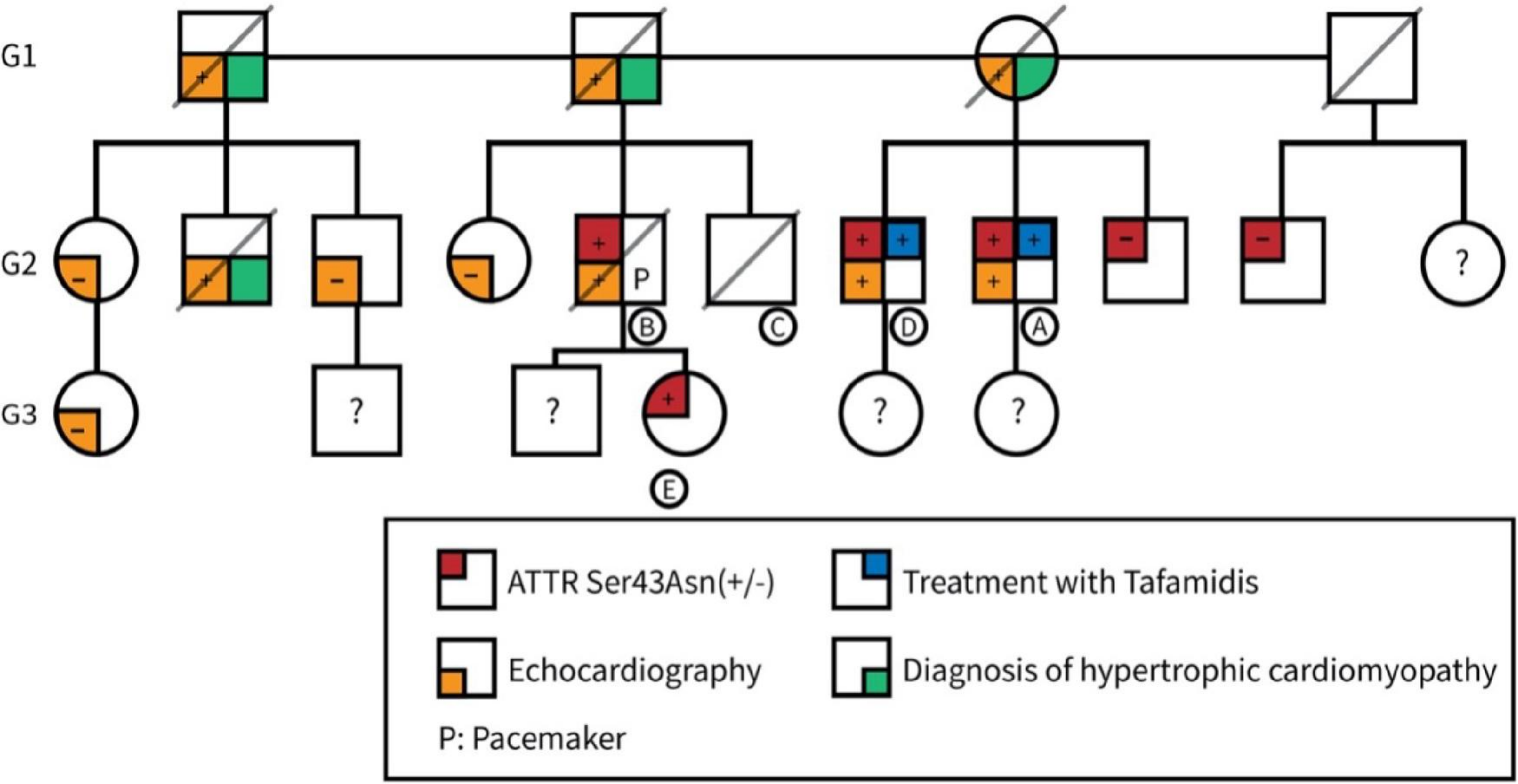
Family tree illustrating the inheritance of the c.128G > A (p.Ser43Asn) mutation.

Patient A's mother had been diagnosed with hypertrophic cardiomyopathy 20 years earlier and died 5 years before his presentation. On the maternal side of the family, four relatives had been diagnosed with hypertrophic cardiomyopathy at different times, including three in the same generation as Patient A's mother and one in the same generation as Patient A; all subsequently died. Patient A's cousin (Patient C) died suddenly at the age of 39 years. Patient C's brother, Patient B, developed exertional dyspnea at 41 years of age and was diagnosed with complete atrioventricular block and recurrent syncope, for which a dual-chamber pacemaker was implanted. Patient B died of refractory heart failure at 43 years of age (Figure 4). Genetic testing performed 1 month before Patient B's death confirmed the presence of the p.Ser43Asn variant in the *TTR* gene.

## Discussion

ATTR–CM is an infiltrative cardiomyopathy that may occur in either wild-type or hereditary forms, with the hereditary form typically inherited in an autosomal dominant manner. In current clinical practice, the diagnosis of ATTR–CM relies primarily on non-invasive radionuclide imaging together with the exclusion of light-chain amyloidosis, whereas *TTR* genetic testing is essential for identifying the hereditary disease subtype (12). Although tissue biopsy with amyloid typing remains the histological gold standard for confirming amyloidosis, current diagnostic algorithms permit a non-biopsy diagnosis of ATTR–CM when bone-avid tracer scintigraphy shows grade 2 or 3 myocardial uptake and monoclonal protein assessment is negative (13, 14).

In the present case, the proband exhibited typical echocardiographic features of cardiac amyloidosis, grade 3 myocardial uptake on [99 m]Tc-PYP scintigraphy with myocardial localization on SPECT/CT, an absence of monoclonal proteins on serum and urine immunofixation electrophoresis, and a heterozygous *TTR* c.128G > A (p.Ser43Asn) variant. These concordant clinical, imaging, laboratory, and genetic findings supported a non-biopsy diagnosis of hereditary ATTR–CM. Although an abdominal fat pad biopsy is less invasive than an endomyocardial biopsy, it is not mandatory when the non-biopsy diagnostic criteria for ATTR–CM are met, and a negative result would not reliably exclude cardiac involvement.

To date, more than 150 *TTR* variants have been identified (15). The p.Ser43Asn variant is a rare *TTR* missense variant initially reported in patients with hereditary transthyretin amyloidosis (16). Subsequent reports described this variant in several patients with ATTR–CM (17–19), and 14 carriers from three unrelated families were later identified in Ecuador (20). Globally, only a limited number of such cases have been documented. Wild-type ATTR remains the most common form overall, whereas Val30Met is one of the most frequently reported variants in hereditary transthyretin amyloidosis. In China, 22 *TTR* variants have been reported (21). Consequently, the present family adds to the limited body of evidence on the p.Ser43Asn variant and expands its known geographic distribution.

Several features of this family deserve attention. First, the disease appeared to have an early onset and a relatively aggressive course. Patient B developed exertional dyspnea at 41 years of age, rapidly progressed to complete atrioventricular block requiring pacemaker implantation, and ultimately died of refractory heart failure. Patient C died suddenly at 39 years of age. Second, the phenotype in this family appeared to be mixed, but cardiac involvement was predominant. Although the proband reported numbness of the left upper limb and decreased superficial sensation, these symptoms occurred in the context of a previous ischemic stroke and were not formally assessed by a neurologist, nerve conduction studies, or a targeted evaluation for carpal tunnel syndrome. Therefore, we could not determine whether these manifestations were related to stroke sequelae, ATTRv-associated peripheral neuropathy, carpal tunnel syndrome, or another neurological condition. Third, the clinical presentation closely resembled hypertrophic cardiomyopathy, which may have contributed to the delayed recognition of ATTR–CM in several affected relatives. However, because most historically affected relatives had died before genetic testing, radionuclide imaging, or tissue typing became available, their previous diagnoses of hypertrophic cardiomyopathy could not be retrospectively verified.

The family history further highlights the phenotypic variability associated with this variant, as well as the limitations of penetrance assessment in this pedigree. Several maternal relatives had previously been diagnosed with hypertrophic cardiomyopathy at relatively young ages based on the clinical information available at that time. However, genetic confirmation, radionuclide imaging, and tissue typing were not available because these relatives had already died. Therefore, their historical diagnoses could not be retrospectively verified. Patient A's mother had also been diagnosed with hypertrophic cardiomyopathy and was the only female relative with documented clinical manifestations in this family. By contrast, Patient E carried the p.Ser43Asn variant but showed no clinical evidence of cardiac or neurologic involvement at the time of evaluation. She declined further assessment for privacy reasons; therefore, her asymptomatic status should be interpreted cautiously and may reflect incomplete or age-dependent penetrance, subclinical disease, or a lack of comprehensive evaluation. Because several affected relatives had died before genetic testing became available and not all at-risk relatives underwent genetic testing or standardized cardiac and neurologic assessments, a formal variant-specific penetrance estimate and a precise age-of-onset distribution could not be determined for this family. In addition, echocardiographic screening in some relatives did not reveal significant structural cardiac abnormalities, although this finding does not exclude subclinical or later-onset disease. Continued cascade genetic screening and longitudinal cardiac and neurologic follow-up will be necessary to clarify the penetrance and full phenotypic spectrum associated with this variant.

The geographic origin of this family also has significance. Previously reported carriers of the p.Ser43Asn variant were mainly from southern Europe and South America, with reports from Asia remaining extremely limited. The present findings, therefore, add to the growing recognition that this rare variant is not confined to a single ethnic group or geographic region. In this respect, the present report complements previous reports from China and other Asian populations and expands the currently available clinical data on this genotype.

More broadly, this case highlights how hereditary ATTR–CM may mimic hypertrophic cardiomyopathy, particularly in the presence of left ventricular wall thickening and a family history of early cardiac death. It also reinforces the importance of considering ATTR–CM in patients with unexplained hypertrophic phenotypes, conduction abnormalities, or discordant clinical features. Early recognition is critical in the era of disease-modifying therapies, enabling both timely intervention and cascade screening to identify additional at-risk relatives before advanced cardiac involvement develops.

Continued follow-up of this family may further clarify the natural history, penetrance, and phenotypic spectrum associated with the *TTR* p.Ser43Asn variant. Identifying additional cases within Asian populations will also be crucial for refining genotype–phenotype correlations and improving the clinical recognition of this rare hereditary ATTR–CM subtype.

### Limitations

This report has several limitations. First, histological confirmation via endomyocardial or extracardiac tissue biopsy was not obtained. However, the diagnosis was supported by concordant non-invasive findings, including grade 3 [99 m]Tc-PYP myocardial uptake, an absence of monoclonal proteins on serum and urine immunofixation electrophoresis, and the identification of a *TTR* pathogenic variant. Second, formal neurological evaluations, nerve conduction studies, and targeted assessment for carpal tunnel syndrome were not performed. Therefore, the presence and extent of ATTRv-associated peripheral nerve involvement could not be fully determined. Third, the pedigree-based penetrance assessment was incomplete. Several affected relatives had died before genetic testing was performed, and not all at-risk relatives underwent genetic testing or standardized cardiac and neurologic evaluation. Therefore, the specific penetrance and age-of-onset distribution of the p.Ser43Asn variant could not be determined for this family. Fourth, the historical diagnoses of hypertrophic cardiomyopathy in deceased relatives could not be retrospectively verified because genetic testing, radionuclide imaging, cardiac magnetic resonance imaging, and tissue confirmation were unavailable for these individuals. Therefore, these historical diagnoses should be interpreted cautiously and cannot be considered definitive evidence of ATTR-CM. Longitudinal follow-up and cascade screening of additional relatives will be essential for clarifying the penetrance, age of onset, and full phenotypic spectrum associated with this variant.

## Conclusion

This case report highlights the rare *TTR* c.128G > A (p.Ser43Asn) variant as a cause of hereditary ATTR–CM in a Chinese family. The clinical presentation mimicked hypertrophic cardiomyopathy and included conduction abnormalities, underscoring the associated diagnostic challenges. Awareness of such atypical presentations is essential for timely diagnosis, appropriate genetic testing, and cascade screening, thereby improving clinical management and informing prognosis for affected individuals.

## Data Availability

The original contributions presented in the study are included in the article/Supplementary Material, further inquiries can be directed to the corresponding author.
